# isiXhosa translation of the Patient Health Questionnaire (PHQ-9) shows satisfactory psychometric properties for the measurement of depressive symptoms [Stage 2]

**DOI:** 10.1177/23982128231194452

**Published:** 2023-08-31

**Authors:** Arish Mudra Rakshasa-Loots, Thandi Hamana, Busiswa Fanqa, Filicity Lindani, Kaylee van Wyhe, Sharon Kruger, Barbara Laughton

**Affiliations:** 1 Family Centre for Research with Ubuntu (FAMCRU), Department of Paediatrics and Child Health, Stellenbosch University, Cape Town, South Africa; 2 Edinburgh Neuroscience, School of Biomedical Sciences, College of Medicine & Veterinary Medicine, The University of Edinburgh, Edinburgh, UK; 3 Division of Biomedical Engineering, Faculty of Health Sciences, University of Cape Town, Cape Town, South Africa; 4 ACSENT Laboratory, Department of Psychology, University of Cape Town, Cape Town, South Africa

**Keywords:** Depressive symptoms, major depressive disorder (MDD), psychometric testing, translation, multicultural tools

## Abstract

Depression is a debilitating illness, and stigma associated with it often prevents people from seeking support. Easy-to-administer and culturally- inclusive tools can allow for early screening for depressive symptoms in primary care clinics, especially in resource-limited settings. In this pre-registered pilot study (Stage 1 Report available at DOI: 10.3389/fpsyt.2022.840912), we produced an open-access isiXhosa-language version of the nine-item Patient Health Questionnaire (PHQ-9), a well-validated measure of depression incidence and severity, using a transcultural translation framework. We validated this isiXhosa PHQ-9 in a sample of *N* = 47 adolescents living with and without HIV in Cape Town, South Africa who speak isiXhosa at home. Reliability, convergent validity, and criterion validity were assessed, with T scores on the Achenbach System of Empirically Based Assessment Youth Self Report (YSR) form completed previously as reference standard. Our isiXhosa PHQ-9 exhibited satisfactory reliability, with Cronbach’s α=0.866 , inter-item correlations ranging from 0.229 to 0.730, and mean item-total correlation of 0.69. PHQ-9 score and Withdrawn/Depressed component T scores on the Youth Self Report were moderately associated (Spearman’s ρ=0.40,p=0.011) , indicating acceptable convergent validity. The isiXhosa PHQ-9 showed satisfactory criterion validity (area under the receiver operating characteristic curve, AUC = 0.706), but these analyses were under-powered. Principal component analysis revealed a one-factor solution, with 45.8% of variance explained by the first principal component and all factor loadings above conventional thresholds. Our isiXhosa translation of the PHQ-9 thus exhibited satisfactory psychometric properties in this pilot validation study and performed comparably to other PHQ-9 versions validated in different languages in African and global contexts. This questionnaire may serve as an invaluable culturally-inclusive screening tool for measuring depressive symptoms among isiXhosa speakers. Caution must be exercised as screening tools including the PHQ-9 may over- or under-estimate prevalence of depression. Further validation in larger, independent cohorts may enable wider use of our isiXhosa PHQ-9 as a screening tool in clinics, research studies, and mental health non-profits who serve amaXhosa.

## Introduction

‘If I show you where I’m struggling, I feel you have been exposed to my weakness’, said Siyanda, a young Xhosa man, in relation to the cultural expectation for Xhosa men to manage mental health issues without seeking support (Williams, 2019: 56). The constellations of behavioural and somatic issues that comprise major depressive disorder (MDD, or depression) are estimated to affect between 4.4% and 5.0% of people globally (Ferrari et al., 2013; World Health Organization (WHO), 2017). Biological and psychosocial factors can predispose individuals to developing MDD (Dobson and Dozois, 2011). Living with a chronic human immunodeficiency virus (HIV) infection, especially, significantly increases the risk of depression (Rezaei et al., 2019), with prevalence of depression among people living with HIV estimated to be as high as 50% (Bhatia and Munjal, 2014; Wang et al., 2018). Given the stigma associated with depression across cultures, people with depression may struggle to access mental healthcare resources, as Siyanda suggests. However, early referral and interventions have substantial benefits for the treatment and prevention of depression (Callander et al., 2017; Rapee, 2013). It is, therefore, imperative to continue to refine tools for early screening of depression and increase their accessibility in primary care clinics.

The Xhosa people (‘amaXhosa’) comprise a diverse cultural group who speak variations of isiXhosa. The vast majority of amaXhosa live in the Eastern Cape and Western Cape provinces of South Africa (Government Communications, 2020). Over the last three centuries, European colonial expansion into the South African heartland occurred at the expense of indigenous populations, including amaXhosa, culminating in systematic disenfranchisement during the apartheid regime (Clark, 2014; Stapleton, 1993). Under the apartheid government, especially, amaXhosa suffered severe marginalisation as Afrikaans was the favoured language in government and education in place of indigenous languages such as isiXhosa (Kaschula, 2008). As a result of these institutional barriers, it is difficult for people to access quality healthcare in isiXhosa today (Levin, 2006; Vergunst et al., 2015). In order to reduce this healthcare inequity faced by amaXhosa, it is crucial that clinical resources are made available to them in their home language.

There is no word for ‘depression’ in isiXhosa, but that does not mean that this debilitating illness is not found among the amaXhosa. In fact, the prevalence of depression in this group, estimated at 6.9%, is significantly higher than global estimates for depression in the general population (Baron et al., 2017). Substantial intra-group variability exists, with, for instance, a significantly higher prevalence rate of 31.9% among women in Khayelitsha during pregnancy and 12 weeks postpartum (Hung et al., 2014). Despite this, few isiXhosa-language tools exist for diagnosis of depression. An early study translated the Edinburgh Postnatal Depression Scale (EPDS) into isiXhosa and found satisfactory internal consistency (De Bruin et al., 2004), but this scale is limited in its target population. Another study validated an isiXhosa version of the Centre for Epidemiological Studies Depression Scale in a more general population group (Baron et al., 2017). The ongoing development and validation of the 16-item South African Depression Scale (SADS) in isiXhosa is particularly promising, especially for amaXhosa living with HIV (Andersen et al., 2020, 2021). However, the trade-off in developing such a tool is that limited comparisons can be made between scores from the SADS and other established scales until it is validated in other countries and cultures, thereby restricting synthesis of results across global studies.

The nine-item Patient Health Questionnaire (PHQ-9) is a useful measure for depression incidence and severity (Kroenke et al., 2001; Kroenke and Spitzer, 2002). It has been validated as a diagnostic tool in clinical samples (Beard et al., 2016) as well as the general population (Martin et al., 2006), where it shows a high degree of convergent validity with other depression scales such as the Beck Depression Inventory (BDI). Furthermore, the PHQ-9 is easy to administer, especially in resource-limited settings (Hancock and Larner, 2009), and exhibits similar results whether it is self-administered or carried out by an interviewer (Pence et al., 2012; Pinto-Meza et al., 2005). It is shown to be useful to screen for depression in African populations (Adewuya et al., 2006) and in people living with HIV (Cholera et al., 2014; Monahan et al., 2009). Numerous validation studies for translations of the PHQ-9 have demonstrated its value as a depression scale in samples across the world (Lotrakul et al., 2008; Mwangi et al., 2020; Woldetensay et al., 2018). Given this global body of evidence, the PHQ-9 represents a measure of depression that could facilitate cross-cultural comparisons of depression pathology better than population- or geography-specific scales such as the EPDS or SADS. Despite these many advantages, to the best of our knowledge, an isiXhosa-language version of the PHQ-9 has not yet been validated and made publicly available. Baron et al. (2017) report findings from an isiXhosa version of the PHQ-9, but this version is not available alongside the study except by request to the authors. Given the utility of the PHQ-9 in rapid screening of depressive symptomatology, a freely available isiXhosa-language PHQ-9 may be an invaluable mental health triage tool for clinicians serving amaXhosa.

In this pilot study, we aimed to produce and validate one of the first open-access isiXhosa-language versions of the PHQ-9 depression scale. The protocol for this study was pre-registered in the Stage 1 Registered Report found at DOI: 10.3389/fpsyt.2022.840912. This isiXhosa PHQ-9 was produced using a transcultural translation framework and administered to a cohort of adolescents living with and without HIV in Cape Town, South Africa. Responses on the PHQ-9 were compared against those on the Achenbach System of Empirically Based Assessment (ASEBA) Youth Self Report (YSR) forms as a reference standard. Our primary hypothesis was that the isiXhosa-language PHQ-9 will exhibit satisfactory reliability, measured as internal consistency using Cronbach’s α . We also hypothesised that this translation of the PHQ-9 will show acceptable convergent validity (correlation coefficient for PHQ-9 and YSR scores) and diagnostic accuracy (area under Receiver Operating Characteristic (ROC) curve (AUC) for PHQ-9 vs YSR). This pilot study aimed to pave the way for larger-scale, independent validation of this isiXhosa-language PHQ-9 and add an easy-to-administer, culturally inclusive questionnaire to the local clinician’s toolbox.

## Methods

### Participants

Participants for this study were recruited through the Adolescent Cognitive and Brain Imaging (GOLD) cohort at Stellenbosch University and Tygerberg Hospital in Cape Town, South Africa. The GOLD study, which draws on the cohort of participants in the landmark Children with HIV Early antiRetroviral therapy (CHER) trial, includes adolescents living with HIV who were initiated on antiretroviral therapy (ART) early in life (Cotton et al., 2013). The CHER cohort is active and regularly willing to contribute to sub-studies, with as many as 80 children living with HIV and 80 age-matched HIV–controls participating in recent sub-studies (Van Biljon et al., 2021).

#### Inclusion criteria

Participants were included in this study if they were younger than 18 years old and spoke isiXhosa at home.

#### Sample size estimation

*A priori* estimation of sample size in validation studies for psychometric tools is remarkably low (Anthoine et al., 2014). For our a priori sample size estimation (described in the Stage 1 Registered Report), we utilised a web-based sample size calculation tool for reliability studies (Arifin, 2018), available at https://wnarifin.github.io/ssc_web.html, with the primary outcome of interest as the Cronbach’s α reliability coefficient for the translated PHQ-9. The *a priori* sample size estimation showed that a sample size of *N* = 19 would be necessary to detect a Cronbach’s α=0.65 (indicative of a moderate reliability) at 80% power for the nine-item questionnaire.

#### Ethical considerations

We received written informed assent from participants and written informed consent from participants’ parents or legal guardians in their language of choice for inclusion in the study before participation. The study was conducted in accordance with the Helsinki Declaration and Good Clinical Practice (GCP) standards. The protocol for this study was approved by the Stellenbosch University Human Research Ethics Committee (N21/10/116_Substudy N19/10/135). Any participants who reported experiencing feelings of self-harm or suicide ideation or scored greater than 15 on the PHQ-9 were referred to a trained psychologist or social worker on staff for psychological support.

### Materials

#### Patient Health Questionnaire

The English-language version of the PHQ-9 was designed with slight adaptations from the original version (Kroenke et al., 2001). The adaptations, intended primarily to improve comprehensibility of the scale to adolescents in 2022, were as follows: in item 7, we replaced ‘reading the newspaper’ with ‘reading’, and in the final question, we replaced ‘if you checked off *any* problems’ with ‘if you chose a number higher than 0’. The isiXhosa-language version of the PHQ-9 was created from this English version using a transcultural translation framework (see Procedure). Both the English-language and isiXhosa-language versions of the PHQ-9 are freely available in Supplemental materials.

#### YSR form

The ASEBA YSR form (Rescorla, 2005) measures behavioural issues representing syndromes such as ‘Withdrawn/Depressed’, ‘thought problems’, and ‘rule-breaking behaviour’. The YSR has been validated as a measure of behavioural issues among adolescents (Ebesutani et al., 2011), including in several studies in southern and eastern Africa (Zieff et al., 2022). In this study, we used data obtained from participants in the GOLD study during previous clinic visits when a validated bilingual (English and isiXhosa) YSR form was administered via an interview by a trained Research Assistant. During these visits, participants were asked (in the language in which they are most comfortable, English or isiXhosa) whether they think they have exhibited any of the behaviours in question over the past 6 months. These responses were used as a reference standard to compare with the translated version of the PHQ-9. T scores for the Withdrawn/Depressed component within Syndrome Scales of the YSR were used to determine participants’ depressive symptoms and classify participants as ‘clinically depressed’, ‘borderline’, or ‘non-depressed’ using ASEBA standards.

### Procedure

#### Setting

The study site for participant recruitment and data collection was the Family Centre for Research with Ubuntu (FAMCRU), Ward J8, Tygerberg Hospital, Department of Paediatrics and Child Health, Faculty of Medicine and Health Sciences, Stellenbosch University.

#### Transcultural isiXhosa translation of the PHQ-9

The process of transcultural translation of psychometric tools involves steps to ensure that translated questionnaires remain accurate, relevant, and culturally acceptable (Kaiser et al., 2013; Kohrt et al., 2016). We adopted this systematic methodology to translate the PHQ-9 into isiXhosa using four steps:

1. Translation from English into isiXhosa by two independent bilingual (English and isiXhosa) speakers;
2. Review of isiXhosa translation by a panel of mental health experts, including research staff and clinical professionals, who speak both languages;
3. Review of translation by a co-production panel involving individuals living with HIV and/or with a lifetime history of depression and who speak both languages; and
4. Blinded back-translation from isiXhosa into English by two additional independent bilingual translators.

The translated version was refined after each step to preserve the core meaning and purpose of each item and incorporate culturally specific idioms describing the affective components measured by the questionnaire where possible. Notably, these refinements were made to accommodate variations in language used by people in different geographical locations and to ensure that the translations were accessible to people who have not received a formal education. Modifications were reconciled in consultation with the translators and review panel involved in this process. The final isiXhosa-language version of the PHQ-9 is available in Supplemental materials.

We also translated a demographic questionnaire, which included inclusive and diverse options for various gender identities and sexual orientations in both English and isiXhosa. This demographic questionnaire is available in Supplemental materials.

#### Co-production

Knowledge co-production, or participatory research methods, involves incorporating insights from individuals with lived experience of the conditions being studied. For this study, we recruited four isiXhosa-speaking individuals as a focus group to review and provide feedback on the isiXhosa translation of the PHQ-9 during the initial stages of this study. Two adults and two adolescents with lived experience of HIV and/or depressive symptoms were recruited through the FAMCRU clinic and the non-profit Inala Mental Health Foundation. These individuals were invited to review the isiXhosa PHQ-9 during Step 3 of the transcultural translation process and suggest edits to improve the accessibility, comprehensibility, and cultural sensitivity of the translation. Co-producers were reimbursed for their time.

#### Participant recruitment and informed consent

Participants were recruited through the GOLD study at the Family Centre for Research with Ubuntu (FAMCRU), Tygerberg Hospital. Adolescents living with or without HIV who met this study’s inclusion criteria were contacted by research staff to ascertain interest in participation in this study. A trained member of the research staff discussed the study procedures in person with the potential participant’s parent or legal guardian in their preferred language. Informed consent and assent forms were available in two versions (English and isiXhosa). Parents or legal guardians of the participants were asked to read and review the consent form. If participants agreed to take part in this study, written informed consent was received from parents or legal guardians in advance of the study procedures and verified by a doctor on staff. All participants were also required to assent to the study procedures. Participants were reimbursed for their travel costs to visit the clinic for the study in line with Stellenbosch University policies.

#### Psychometric testing

Participants were provided a private space for psychometric testing. A member of the research staff briefed each participant on the procedures involved in the study and receive informed assent. The research staff confirmed that the participant would prefer to respond to questionnaires in isiXhosa and offered them a choice between filling out the questionnaire themselves in writing or having the research staff ask them the questions verbally (for participants who may not be able to write or have a preference for spoken isiXhosa over written isiXhosa). If participants chose to complete the psychometric testing in writing, they were asked to fill out a short demographic questionnaire and the translated version of the PHQ-9 in writing (paper and pencil). If participants chose to complete the questionnaire verbally, the member of the research staff read each item on the questionnaire exactly as written in a neutral manner and wrote down the participant’s response, without judgement or comment. In either case, the research staff member was available to the participant for any clarifications to ensure participants understood what each question was asking. Once participants completed the demographic questionnaire and the PHQ-9, the research team member verified that all nine items of the PHQ-9 were completed. The research team member calculated the total score on the PHQ-9 as the sum of the scores from individual items and input responses into a secure electronic database (Project RedCap), which included built-in quality control checks. Data were handled in accordance with the Protection of Personal Information Act (South Africa) and the UK General Data Protection Regulation (GDPR) Act.

### Statistical approach

All statistical analyses were performed using R version 4.2.1. Participant demographic characteristics were summarised using proportions (*n* and %) for categorical variables, and medians and interquartile ranges (IQRs) for continuous variables.

To determine the reliability of the isiXhosa PHQ-9, we calculated Cronbach’s α as a measure of internal consistency for the translated version. Inter-item (excluding correlation of an item with itself) and item-total score correlations were also calculated. To determine convergent validity for the PHQ-9, Spearman’s correlation coefficients were calculated for the total PHQ-9 scores and T scores for the Anxious/Depressed and Withdrawn/Depressed syndrome scales on the YSR. To determine the criterion validity of this PHQ-9 version, we compared participant responses on the isiXhosa PHQ-9 with the Withdrawn/Depressed scale on YSR forms as a reference standard, to calculate diagnostic sensitivity and specificity, predictive values (PPV/NPV), likelihood ratios (PLR/NLR), and Youden’s Index. Participants with a Withdrawn/Depressed T score in borderline (65≤T≤69) or clinical (T≥70) range were assigned values of 1 (representing cases), and all other participants (T<65) were assigned values of 0 (representing controls). An ROC curve was produced and AUC for the PHQ-9 was calculated to assess diagnostic performance in comparison to the YSR. Finally, individual item analyses were conducted to determine whether mean scores on each item of the PHQ-9 differed between participants who met clinical or borderline depression thresholds and those who did not, as categorised by withdrawn/depressed T scores on the YSR. For item-level analyses, that is, comparing mean scores between the isiXhosa PHQ-9 and a global pooled sample and comparing mean scores between participants classified using the YSR as having Borderline or Clinical Depression and those having No Depression, *p*-values were corrected using the false discovery rate (FDR) method to adjust for multiple comparisons.

### Deviations from Stage 1 Registered Report

Certain deviations from the protocol described in the Stage 1 Registered Report are noted. First, the Stage 1 Registered Report indicated that only adolescents living with HIV would be recruited. However, given that the validity of the PHQ-9 is not specific to this population and to ensure the maximum possible power for our analyses, we included both adolescents living with and without HIV in this study. Second, the Stage 1 Registered Report indicated that the PHQ-9 and YSR scores would be separated by no more than 3 months, but this was not possible due to practical constraints in participant scheduling. We, therefore, report the median absolute time difference between the YSR and PHQ-9 administration. In all cases, the most recent YSR scores available for each participant were included for analysis, and the YSR and PHQ-9 were administered as closely as possible, as was indicated in the registered protocol. To ensure that these deviations from the registered protocol did not substantially impact our findings, we conducted sensitivity analyses, which are described below.

### Exploratory analyses

In addition to pre-registered analyses described above, we carried out descriptive analyses of group differences, as our sample included both participants with and without HIV. Group differences in demographic characteristics were assessed using Pearson’s chi-square test or Fisher’s exact test for categorical variables, and Wilcoxon rank sum test for continuous variables. Group differences were assessed by HIV status, comparing participants with HIV and participants without HIV, and depressive symptom status, comparing participants with total PHQ-9 score >4 ( ‘Some Depressive Symptoms’) and those with score ≤4 ( ‘Minimal Depressive Symptoms’; Kroenke et al., 2001).

We also investigated the construct validity and factor structure of our isiXhosa-language PHQ-9. We conducted a principal component analysis (R function principal with varimax rotation) to calculate eigenvalues and factor loadings for each item on the questionnaire. A scree plot was produced using variance explained for each component to confirm number of factors. Tucker’s coefficient of factor congruence was calculated to assess the congruence between factor loadings in our sample with other reported PHQ-9 validation studies. To ascertain whether the variability in factor loadings for each item in our translation may be attributed to the small sample size in our pilot study, a bootstrap analysis was run (R function ‘boot’) with 1000 sets of bootstrapped factor loadings based on our observed factor loadings. Using the bootstrap output, bias corrected accelerated 95% confidence intervals (CIs; BC α , R function boot.ci, type = ‘bca’) were calculated for all nine items.

To demonstrate the comparability of our isiXhosa-language PHQ-9 with other versions of the PHQ-9, in addition to describing the inherent psychometric properties of our translation, we also present comparisons with published psychometric properties of other PHQ-9 versions in African and global contexts. These include a seTswana version in South Africa (Bhana et al., 2015), an English version in Kenya (Monahan et al., 2009), a Portuguese version in Mozambique (Cumbe et al., 2020), a Swahili version in Tanzania (Fawzi et al., 2019), and a recent multi-country, multi-language validation study (Bianchi et al., 2022).

#### Sensitivity analyses for deviations from Stage 1 Registered Report

To ensure that the inclusion of participants without HIV in our study did not unduly influence our findings, we conducted sensitivity analyses for reliability and construct validity, following the same procedures as above, for the subset of participants with HIV only. To ensure that greater time difference between PHQ-9 and YSR administration did not unduly influence our findings, we conducted sensitivity analyses for convergent validity using partial Spearman’s correlation coefficients, which controlled for time between PHQ-9 and YSR administration. We also conducted sensitivity analyses for criterion validity with the same procedures as above, for only the subset of participants for whom absolute time difference between PHQ-9 and YSR administration was less than 100 days.

## Results

### Participant characteristics

We included *N* = 47 participants in this pilot validation study (see Table 1). Median (IQR) age of participants was 16 (15, 16) years. Participants were relatively evenly split by gender (47% girls), $\chi^{2}\left(1\right)=0.20,p=0.662$ . All participants were currently enrolled in school, ranging in grade from Grade 7 to Grade 11. All participants identified as Black/African (*n* = 47, 100%) and most identified as heterosexual $\left(n=44,94\%,\chi^{2}\left(2\right)=76.9p<0.001\right)$ . Participants living with HIV (*n* = 35, 74.5%) were all receiving ART, and 91.4% were virally-suppressed (viral load <200 copies/μL; see Table 2).

**Table 1.** Demographic characteristics for all participants included in the study, and for subgroups by HIV status and depressive symptoms.

Characteristic	Overall *N* = 47	Participants with HIV *n* = 35	Participants without HIV *n* = 12	*p*-value	Some depressive symptoms *n* = 16	Minimal depressive symptoms *n* = 31	*p*-value
Age (years), Median (IQR)^a^	16.0 (15.0, 16.0)	16.0 (15.0, 16.0)	14.5 (14.0, 15.2)	**0.006**	16.0 (15.0, 16.0)	16.0 (15.0, 16.0)	> 0.9
Grade in School, *n* (%)^b^				0.100			0.4
7	3 (6.4)	1 (2.9)	2 (17)		2 (12)	1 (3.2)	
8	8 (17)	6 (17)	2 (17)		4 (25)	4 (13)	
9	11 (23)	6 (17)	5 (42)		2 (12)	9 (29)	
10	20 (43)	17 (49)	3 (25)		6 (38)	14 (45)	
11	5 (11)	5 (14)	0 (0)		2 (12)	3 (9.7)	
Gender, *n* (%)^b^				**0.015**			0.3
Boy	25 (53)	15 (43)	10 (83)		7 (44)	18 (58)	
Girl	22 (47)	20 (57)	2 (17)		9 (56)	13 (42)	
Ethnicity, *n* (%)^b^							> 0.9
Black/African	47 (100)	35 (100)	12 (100)		16 (100)	31 (100)	
Sexual orientation, *n* (%)^b^				> 0.9			0.3
Bisexual/Pansexual	1 (2.1)	1 (2.9)	0 (0)		1 (6.2)	0 (0)	
Gay/Lesbian/Homosexual	2 (4.3)	2 (5.7)	0 (0)		1 (6.2)	1 (3.2)	
Straight/Heterosexual	44 (94)	32 (91)	12 (100)		14 (88)	31 (97)	
Alcohol use (yes), *n* (%)^c^	10 (21)	6 (17)	4 (31)	0.4	3 (19)	7 (23)	> 0.9
Cigarette smoking (yes), *n* (%)^c^	2 (4.3)	2 (5.7)	0 (0)	> 0.9	1 (6.2)	1 (3.2)	> 0.9
Recreational drug use (yes), *n* (%)^c^	5 (11)	5 (14)	0 (0)	0.3	4 (25)	1 (3.2)	0.037

‘Some Depressive Symptoms’ indicates PHQ-9 score >4 , and ‘Minimal Depressive Symptoms’ indicates PHQ-9 score ≤4. Bold text indicates a *p* value less than 0.05.

HIV: human immunodeficiency virus; IQR: interquartile range.

a Wilcoxon rank sum test.

b Pearson’s chi-square test.

c Fisher’s exact test.

**Table 2.** HIV-related clinical characteristics for participants living with HIV included in the study.

HIV-related clinical characteristic	Participants with HIV, *n* = 35
Viral load < 200 copies/μL, *n* (%)	32 (91.4)
Absolute CD4 count, median (IQR)	788.5 (622.2, 987.2)
(Missing)	3
Antiretroviral regimen, *n* (%)	
ABC/3TC combination tablets	1 (2.9)
Atazanavir	3 (8.6)
DTG	1 (2.9)
F/TAF	1 (2.9)
Lopinavir + Ritonavir (LPV/r, Kaletra)	3 (8.6)
TLD combination tablets	26 (74)

HIV: human immunodeficiency virus; IQR: interquartile range.

Demographic characteristics by subgroups of HIV status and depressive symptoms are reported in Table 1. The majority of participants were adolescents living with HIV (*n* = 35, 74.5%), although as the utility of the PHQ-9 as a screening tool for depressive symptoms is not limited to people living with HIV, we did include participants without HIV (*n* = 12, 25.5%) in this study. Participants did not differ on demographic characteristics when stratified by HIV status, except participants with HIV were slightly older, and a greater proportion of participants without HIV were boys.

Median (interquartile range (IQR)) total score on the PHQ-9 was 3 (0, 7), with a range of 0–17. Of the full sample, *n* = 16 (34%) participants met the criterion for ‘some depressive symptoms’ (total PHQ-9 score > 4). Participants did not differ on demographic characteristics when stratified by depressive symptoms, except participants with some depressive symptoms were more likely to report having used recreational drugs in the past 6 months.

Median (IQR) absolute time difference between administration of the PHQ-9 and YSR was 92 (52, 131) days.

### Reliability

**Figure 1.**
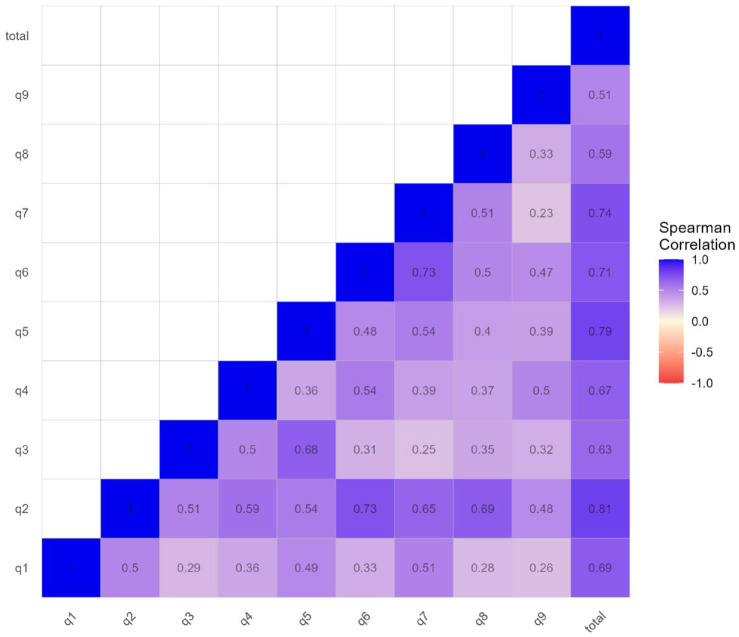
Inter-item correlations for the isiXhosa PHQ-9. Spearman’s rank correlation coefficients (ρ) were determined using pairwise complete observations in our sample. Strength of correlations are graded across a 3-point scale: –1.0 (in deep red), 0.0 (in light yellow), and +1.0 (in deep blue).

Cronbach’s α for our isiXhosa PHQ-9 was 0.866, suggesting high internal consistency. Inter-item correlations ranged from 0.229 to 0.730 (see Figure 1). Mean (standard deviation (SD)) scores on each item, and mean inter-item correlations (excluding correlations of an item with itself) are shown in Table 3. Mean item-total correlation was 0.688.

**Table 3.** Mean score with standard deviation (SD) and inter-item correlation for each item of the isiXhosa PHQ-9.

Question	Item	Mean	SD	Inter-item correlations
q1	Anhedonia	0.617	0.765	0.376
q2	Depressed mood	0.574	0.950	0.587
q3	Sleep changes	0.532	0.929	0.401
q4	Loss of energy	0.426	0.542	0.451
q5	Appetite changes	0.702	1.082	0.483
q6	Low self-esteem	0.404	0.712	0.511
q7	Trouble concentrating	0.468	0.687	0.476
q8	Psychomotor changes	0.298	0.587	0.428
q9	Suicide ideation	0.191	0.537	0.371

SD: standard deviation.

**Figure 2.**
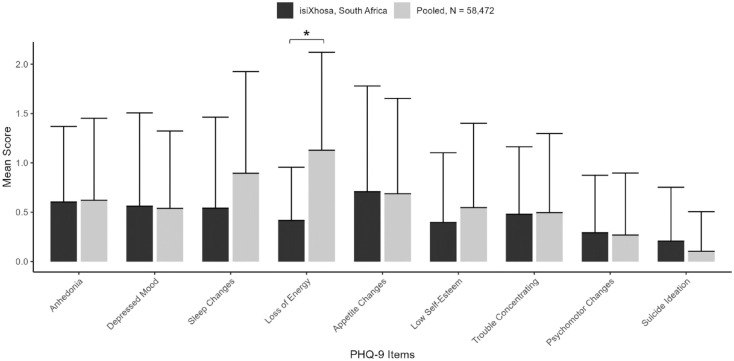
Mean scores on each item of the PHQ-9. Mean scores are compared between our isiXhosa PHQ-9 and means from a large pooled sample reported by Bianchi et al. (2022). Error bars depict standard error of the mean (SEM). ^*^Indicates a significant difference in group means (Welch’s *t*-test, FDR-corrected *p* < 0.05).

Item-level mean scores for our isiXhosa PHQ-9 were comparable to those from a large (*N* = 58,472) validation study recently carried out, which reported PHQ-9 data pooled from participants in seven countries (France, Germany, Israel, New Zealand, Spain, Switzerland, and the United States) and administered in five languages (French, German, English, Hebrew, Spanish; Bianchi et al., 2022; see Figure 2). Welch’s *t*-tests were used to compare means between the two data sets, which revealed that mean scores on only Question 4 (loss of energy) differed significantly between our data set and the larger pooled validation data set, *t*(29) = –7.11, $P_{FDR}<0.001$ (all other $p_{FDR}>0.05$ ). Mean score on Question 4 in our sample (*M* = 0.43, *SD* = 0.54) was lower than that in the sample of Bianchi et al. (*M* = 1.13, *SD* = 0.99).

In all, our isiXhosa PHQ-9 exhibited satisfactory reliability, and item-level scores were comparable to previously published findings.

### Convergent validity with the YSR

There was a moderate significant association between total PHQ-9 score and T score on the Withdrawn/Depressed component of the YSR (Spearman’s ρ=0.44 , *p* = 0.002). The correlation between total PHQ-9 score and T score on the Anxious/Depressed component of the YSR was not significant (Spearman’s ρ=0.121 , *p* = 0.419). Thus, the isiXhosa PHQ-9 demonstrated acceptable convergent validity with the Withdrawn/Depressed component of the YSR, but not with the Anxious/Depressed component.

### Criterion validity

**Figure 3.**
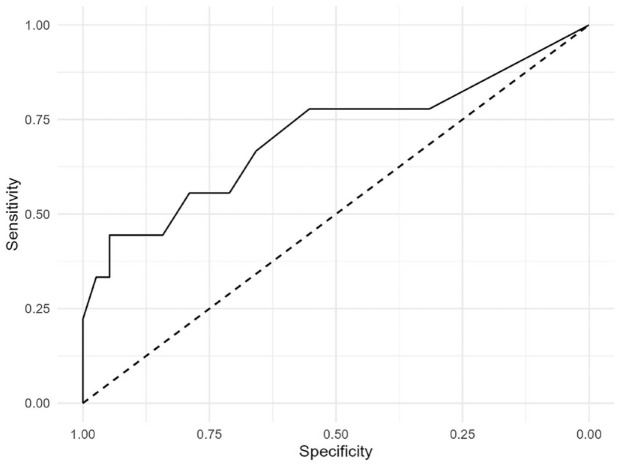
Receiver Operating Characteristic (ROC) curve for the isiXhosa PHQ-9. Area under the ROC curve (AUC) with 95% confidence intervals was 0.671 (0.400, 0.942).

**Figure 4.**
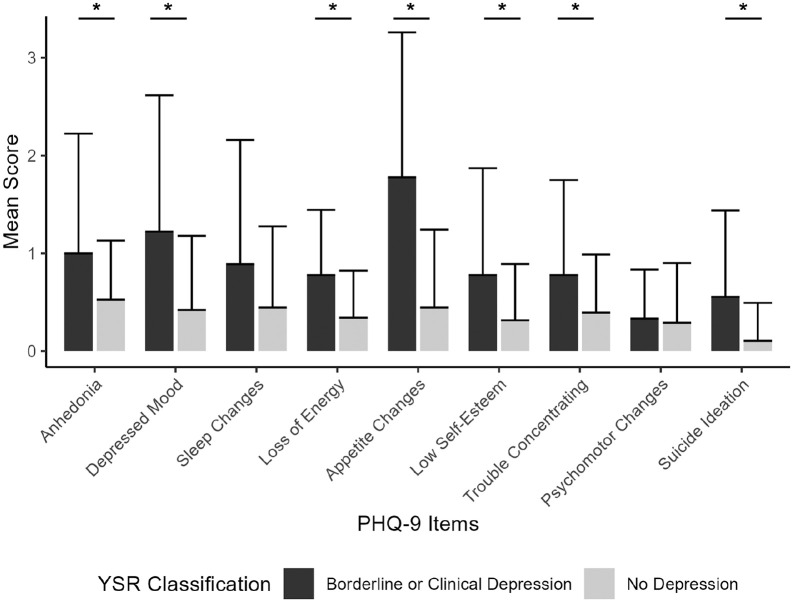
Mean scores on each item of the isiXhosa PHQ-9 by withdrawn/depressed classification on the YSR. Participants with borderline or clinical T scores on the YSR were pooled. Error bars depict standard error of the mean (SEM). ^*^Indicates FDR-corrected *p*-value < 0.05.

Using T score thresholds, *n* = 5 participants met borderline criteria and *n* = 4 met clinical criteria on the Withdrawn/Depressed YSR component. The ROC curve for the isiXhosa PHQ-9 is shown in Figure 3. Area under the ROC curve (AUC) (95% CIs) was 0.706 (0.476, 0.937), indicating acceptable discrimination. Characteristics of the ROC curve are shown in Table 4. A threshold of PHQ-9 score ≥10 provided satisfactory specificity of 94.74% but poor sensitivity of 44.44%, with a Youden’s Index of 1.39. Item-level analyses (see Figure 4) indicated that participants with a borderline or clinical T score on the Withdrawn/Depressed component of the YSR scored significantly higher on most items of the isiXhosa PHQ-9 $\left(p_{FDR}<0.05\right)$ , except on questions about sleep changes $\left(p_{FDR}=0.055\right)$ and psychomotor changes $\left(p_{FDR}=0.704\right)$ .

**Table 4.** Diagnostic performance of the isiXhosa PHQ-9 in comparison with the Youth Self Report form as a reference standard.

Threshold	Sensitivity %	Specificity %	PPV	NPV	PLR	NLR	1-Sensitivity	1-Specificity	Youden’s Index
≥1	77.78	31.58	0.21	0.86	1.14	0.70	0.22	0.68	1.09
≥2	77.78	44.74	0.25	0.89	1.41	0.50	0.22	0.55	1.23
≥3	77.78	55.26	0.29	0.91	1.74	0.40	0.22	0.45	1.33
≥4	66.67	65.79	0.32	0.89	1.95	0.51	0.33	0.34	1.32
≥5	55.56	71.05	0.31	0.87	1.92	0.63	0.44	0.29	1.27
≥6	55.56	73.68	0.33	0.88	2.11	0.60	0.44	0.26	1.29
≥7	55.56	78.95	0.38	0.88	2.64	0.56	0.44	0.21	1.35
≥8	44.44	84.21	0.40	0.86	2.81	0.66	0.56	0.16	1.29
≥9	44.44	89.47	0.50	0.87	4.22	0.62	0.56	0.11	1.34
≥10	44.44	94.74	0.67	0.88	8.44	0.59	0.56	0.05	1.39
≥11	33.33	94.74	0.60	0.86	6.33	0.70	0.67	0.05	1.28
≥14	33.33	97.37	0.75	0.86	12.67	0.68	0.67	0.03	1.31
≥16	22.22	100.00	1.00	0.84	Inf	0.78	0.78	0.00	1.22

PPV: positive predictive value, NPV: negative predictive value, PLR: positive likelihood ratio, NLR: negative likelihood ratio.

Due to the small number of participants meeting criteria for borderline or clinical depression as indicated by the YSR, analyses of criterion validity were under-powered. A *post hoc* power analysis estimated that the AUC of 0.706 with 9 cases and 38 controls was detected with only 50% power. Therefore, the isiXhosa PHQ-9 demonstrated acceptable criterion validity against the YSR as a reference standard, but these analyses were under-powered and warrant replication in a larger sample.

### Exploratory analyses: construct validity

**Figure 5.**
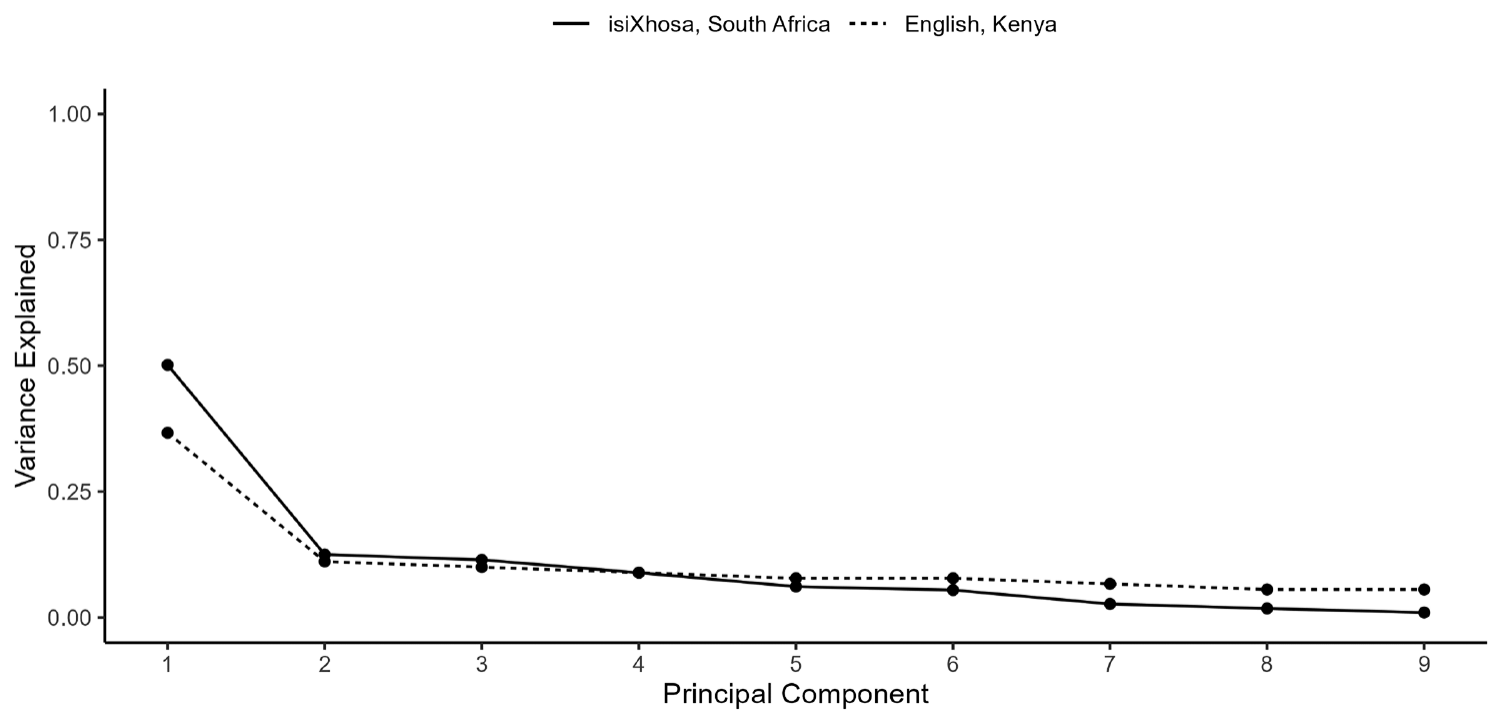
Scree plot of variance explained by each principal component of the PHQ-9. Variances explained are plotted for our isiXhosa PHQ-9 and for an English version validated in Kenya Monahan et al. (2009) for comparison.

Given previous findings in the literature, we expected to see a one-factor solution for our isiXhosa PHQ-9. The scree plot (see Figure 5) indicated one dominant dimension with a substantial decrease in eigenvalues between the first and second principal component, and small decreases thereafter (eigenvalues: 4.51, 1.12, 1.03, 0.80, 0.55, 0.49, 0.24, 0.16, and 0.09). The proportion of variance explained by the first principal component was 49.6%. The scree plot of variance explained for our isiXhosa PHQ-9 was comparable to that for Monahan et al. (2009).

**Figure 6.**
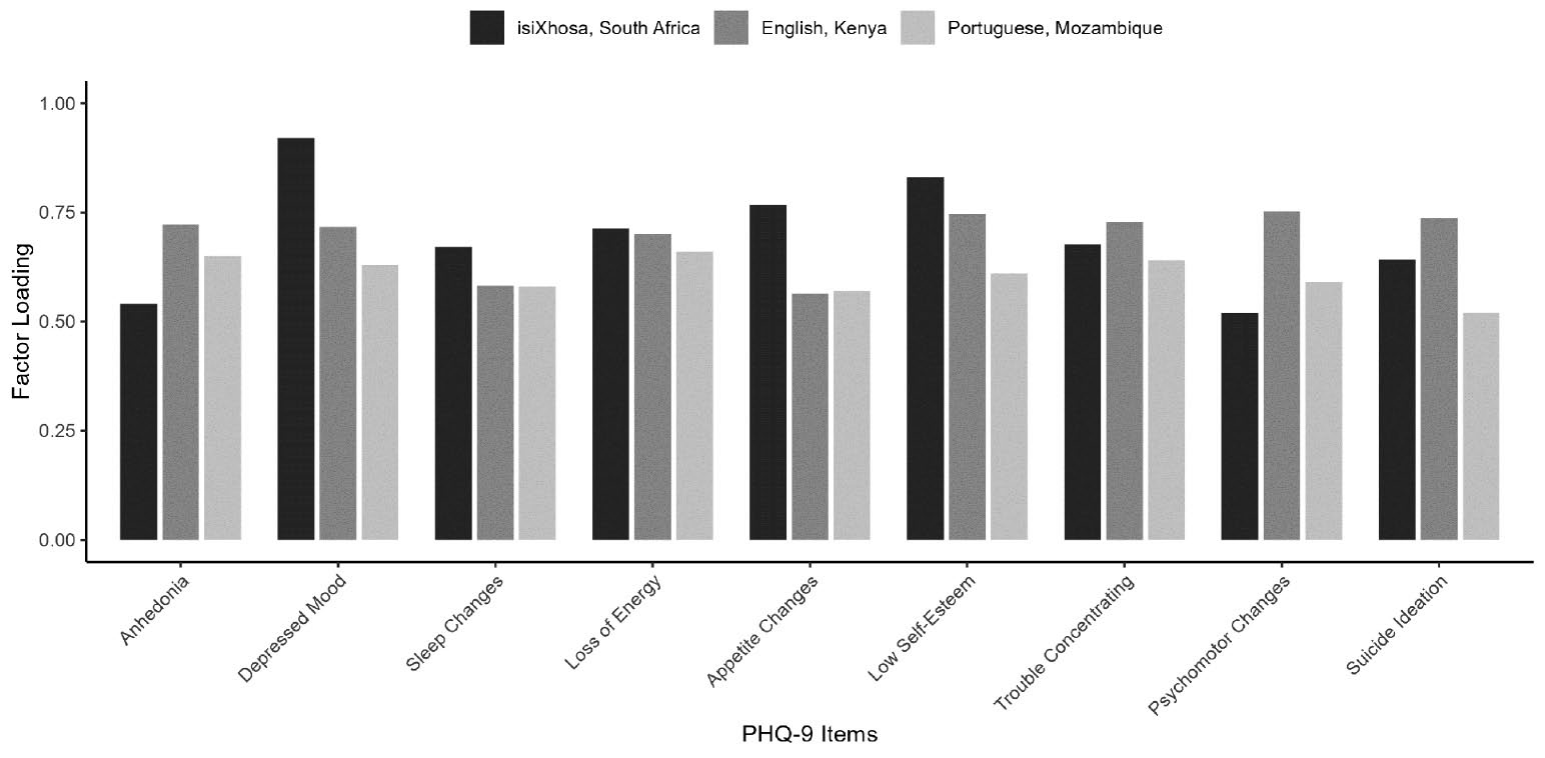
Factor loadings for each item of the PHQ-9. Factor loadings are plotted for our isiXhosa PHQ-9, an English version validated in Kenya Monahan et al. (2009), and a Portuguese version validated in Mozambique Cumbe et al. (2020).

Factor loadings ranged from 0.52 to 0.92 (thus exceeding the 0.40 cut-off (Howard, 2016)) and item-total correlations ranged from 0.51 to 0.81 (thus exceeding the 0.30 cut-off; see Table 5). Factor loadings across various items of the isiXhosa PHQ-9 were more variable (i.e. had a larger range) than observed in other versions of the PHQ-9 (see Figure 6). However, Tucker’s coefficients of factor congruence indicated high congruence between factor loadings in our sample and those reported by Monahan et al. (2009) (ϕ=0.98) and Cumbe et al. (2020) (ϕ=0.98) . To determine whether this variability in factor loadings may be attributed to our smaller sample size, we calculated 95% CIs with 1000 sets of bootstrapped factor loadings for all nine items. For most items, with the exception of Question 2 (depressed mood), the factor loadings reported in at least one of these two previous studies fell within the CIs of the bootstrap realisations, suggesting that the variability in factor loadings for our version may have arisen due to the small sample size of our pilot study (Supplemental materials). Overall, we observed that our isiXhosa PHQ-9 demonstrated a one-factor structure with satisfactory factor loadings and item-total correlations.

**Table 5.** Factor loadings and item-total correlations for each item of the isiXhosa PHQ-9.

Question	Item	Factor loading	Item-total correlations
q1	Anhedonia	0.54	0.69
q2	Depressed mood	0.92	0.81
q3	Sleep changes	0.67	0.63
q4	Loss of energy	0.71	0.67
q5	Appetite changes	0.77	0.79
q6	Low self-esteem	0.83	0.71
q7	Trouble concentrating	0.68	0.74
q8	Psychomotor changes	0.52	0.59
q9	Suicide ideation	0.64	0.51

### Sensitivity analyses

To assess whether the inclusion of participants without HIV in this study may have influenced our findings, we conducted sensitivity analyses for the subset of participants with HIV (*n* = 35) only. Sensitivity analyses of reliability showed that the Cronbach’s α for participants with HIV only was 0.861. Mean item-total correlation was 0.640. Mean (standard deviation (SD) scores on each item, and mean inter-item correlations (excluding correlations of an item with itself), were comparable to the full sample and are available in Supplemental materials. Sensitivity analyses of construct validity using principal component analysis indicated one dominant dimension, with 49.0% of variance explained by the first principal component, thus comparable to the full sample. Eigenvalues, factor loadings, and scree plot of variance explained for this subset are available in Supplemental materials. In all, reliability and construct validity of the isiXhosa PHQ-9 was comparable among participants with HIV only and the full sample.

To assess whether the greater time difference between PHQ-9 and YSR administration may have influenced our findings, we conducted further sensitivity analyses. For convergent validity, we calculated partial Spearman’s correlation coefficients adjusted for absolute time difference between the two assessments. Findings remained consistent, with a moderate significant association between total PHQ-9 score and withdrawn/depressed T score (Spearman’s ρ=0.45 , *p* = 0.002), but no significant correlation between total PHQ-9 score and anxious/depressed T score (Spearman’s ρ=0.12 , *p* = 0.417). Finally, we assessed criterion validity in only the subset of participants for whom the PHQ-9 and YSR were administered less than 100 days apart (*n* = 25). Of this subset, *n* = 6 participants met criteria for clinical or borderline depression on the YSR. The ROC curve and diagnostic performance characteristics for this subset are available in Supplemental materials. AUC (95% CIs) was 0.79 (0.54, 1.00), indicating satisfactory discrimination. A threshold of PHQ-9 score ≥7 yielded 84.2% sensitivity and 66.7% sensitivity, with Youden’s Index of 1.51. Therefore, diagnostic performance of the isiXhosa PHQ-9 was slightly better among participants for whom the YSR and PHQ-9 were administered closer together.

## Discussion

In this Stage 2 Registered Report, we produced an isiXhosa translation of the PHQ-9 and assessed its psychometric properties. Crucially, we have demonstrated that our isiXhosa PHQ-9 exhibits psychometric properties comparable to those of other PHQ-9 versions in Africa and beyond. Internal consistency (α=0.864) observed for our isiXhosa PHQ-9 is compatible with previous reports of Cronbach’s α of 0.76 (South Africa (Bhana et al., 2015)), 0.78 (Kenya (Monahan et al., 2009)), and 0.83 (Tanzania (Fawzi et al., 2019)) for the PHQ-9 in other African contexts. Similarly, the factor structure of our isiXhosa PHQ-9 was comparable with other versions in African nations (Kenya (Monahan et al., 2009) and Mozambique (Cumbe et al., 2020)). Overall, we have shown that our isiXhosa PHQ-9 performs comparably to other versions, which supports the adoption of this translation into research and clinics.

Item-level mean scores in our study were comparable with the large, global sample reported by Bianchi et al. (2022). We only observed a significant difference in mean score on Question 4, concerning fatigue and loss of energy, which participants in our study reported less frequently than participants in the pooled sample. One possible explanation for this difference may be an effect of age. While our study included only adolescents (median age 16 years), the pooled sample included primarily older adults (mean age 43 years), with many non-adolescent participants even in the youngest subgroup (private correspondence with Dr Bianchi). Older participants may be more likely to report fatigue and loss of energy, which may explain the difference in mean score between the two samples.

A key limitation of this study is the small sample size and low prevalence of depressive symptoms. Despite the increased overall power afforded to the study by our larger-than-planned sample size, the proportion of participants who were classified as ‘clinically depressed’ or ‘borderline depressed’ on the YSR was extremely small. As a result, assessments of criterion validity were underpowered. Larger validation studies in future are necessary to assess criterion validity of this isiXhosa PHQ-9 with sufficient power. Another potential limitation is that the diagnostic performance of our isiXhosa PHQ-9 was compared against the YSR as a reference standard, rather than a clinical diagnostic interview, and that the PHQ-9 and YSR were not administered on the same day. The YSR has been shown to predict a ‘true’ diagnosis of depression (i.e. determined using an interview by a psychiatrist) with acceptable sensitivity and specificity (Geibel et al., 2016). Nevertheless, future studies may assess the criterion validity of the isiXhosa PHQ-9 against a clinical interview rather than a screening tool such as the YSR.

However, it must be noted that the PHQ-9 itself is a screening tool, which may support triage for people experiencing depressive symptoms, before a comprehensive diagnostic evaluation by a clinician is carried out. Although defining a cut-off on the PHQ-9 can enable putative diagnosis of MDD, this questionnaire is not in fact sufficient for diagnosis. More broadly, measurement of depressive symptoms – including using screening tools such as the PHQ-9 – suffers from substantial methodological and theoretical challenges, which impair our ability to accurately detect depression. (Fried et al., 2022) Depending on the cut-off chosen (and there is no universal consensus on the ‘correct’ cut-off), the PHQ-9 may severely under- or over-estimate the prevalence of depression. (A simulation of the extent of this error may be found at http://depressionscreening100.com/phq/.) Moreover, measurement of depressive symptoms on a continuous scale, rather than categorical incidence of MDD, can be useful in identifying symptom clusters, tracking subtle changes in symptom severity, or assessing efficacy of antidepressant interventions beyond a strict diagnostic binary. For these reasons, there is intrinsic value in the assessment of the utility of the isiXhosa PHQ-9 as a screening tool for a continuous spectrum of depressive symptoms, and in comparison with other screening tools, rather than an over-emphasis on comparisons with clinical diagnostic tools.

At the time of registration of our Stage 1 Registered Report, there was no openly available isiXhosa translation of the PHQ-9. Since then, another research group has independently produced a similar isiXhosa-language PHQ-9, which merits discussion. Marlow et al. (2023) also used a transcultural translation framework to produce their version of the PHQ-9. They then assessed the criterion validity of their PHQ-9 in comparison with the Kiddie Schedule for Affective Disorders and Schizophrenia (K-SADS) in a sample of 302 adolescents in South Africa. They reported high discrimination (AUC = 0.88) for their overall sample when comparing their PHQ-9 with the K-SADS. Given that translation of the PHQ-9 by Marlow et al. was produced with a similar methodology and with comparable adjustments to the original English as our isiXhosa PHQ-9, these independent results lend further support to the utility of the isiXhosa PHQ-9. The study by Marlow et al benefits from a much larger sample size than ours, suggesting that their analyses of criterion validity may be more sufficiently powered. Conversely, our study offers the advantage of assessing additional psychometric properties (beyond criterion validity) for the isiXhosa PHQ-9, including reliability, convergent validity with the YSR, and factor structure. In addition, our study and statistical analysis plans were pre-registered to minimise any risk of bias towards positive results. Together, our study and that by Marlow et al. independently support the utility of the isiXhosa PHQ-9 as a tool for measuring the incidence and severity of depressive symptoms.

isiXhosa is a primarily oral language, and many amaXhosa (particularly young people) are more comfortable with spoken isiXhosa than written isiXhosa. In keeping with this, many of our adolescent participants requested that the isiXhosa PHQ-9 be read aloud to them. Assessments of emotional or behavioural states may be most accurate when conducted in participants’ home language and in a format in which they are most comfortable. We have, therefore, identified the need to standardise and optimise this process. One solution is to produce an audio-recorded version of the isiXhosa PHQ-9, which may be administered digitally (in an app format) with recordings playing alongside the text of the questions. In future, we hope to produce this app-based version of the isiXhosa PHQ-9, which may allow access to this screening tool for participants who can speak (but not read) isiXhosa, as well as reducing staff burden and minimising inter-interviewer variability.

In this pilot study, we have demonstrated that our isiXhosa translation of the PHQ-9 exhibits satisfactory psychometric properties. Larger, independent validation studies are necessary to further characterise the diagnostic performance of this isiXhosa PHQ-9 as our analysis of criterion validity was under-powered, although the study by Marlow et al. (2023) using a similar translation suggests that the isiXhosa PHQ-9 may show acceptable diagnostic performance. Given the brevity and ease of administration of the PHQ-9, and the transcultural translation framework, which we used to produce this translation, our isiXhosa PHQ-9 may be an invaluable tool for measuring depressive symptoms in clinics, research groups, and mental health non-profits serving isiXhosa speakers.

## Supplemental Material

sj-csv-5-bna-10.1177_23982128231194452 – Supplemental material for isiXhosa translation of the Patient Health Questionnaire (PHQ-9) shows satisfactory psychometric properties for the measurement of depressive symptoms [Stage 2] Click here for additional data file. Supplemental material, sj-csv-5-bna-10.1177_23982128231194452 for isiXhosa translation of the Patient Health Questionnaire (PHQ-9) shows satisfactory psychometric properties for the measurement of depressive symptoms [Stage 2] by Arish Mudra Rakshasa-Loots, Thandi Hamana, Busiswa Fanqa, Filicity Lindani, Kaylee van Wyhe, Sharon Kruger and Barbara Laughton in Brain and Neuroscience Advances

sj-csv-6-bna-10.1177_23982128231194452 – Supplemental material for isiXhosa translation of the Patient Health Questionnaire (PHQ-9) shows satisfactory psychometric properties for the measurement of depressive symptoms [Stage 2] Click here for additional data file. Supplemental material, sj-csv-6-bna-10.1177_23982128231194452 for isiXhosa translation of the Patient Health Questionnaire (PHQ-9) shows satisfactory psychometric properties for the measurement of depressive symptoms [Stage 2] by Arish Mudra Rakshasa-Loots, Thandi Hamana, Busiswa Fanqa, Filicity Lindani, Kaylee van Wyhe, Sharon Kruger and Barbara Laughton in Brain and Neuroscience Advances

sj-csv-7-bna-10.1177_23982128231194452 – Supplemental material for isiXhosa translation of the Patient Health Questionnaire (PHQ-9) shows satisfactory psychometric properties for the measurement of depressive symptoms [Stage 2] Click here for additional data file. Supplemental material, sj-csv-7-bna-10.1177_23982128231194452 for isiXhosa translation of the Patient Health Questionnaire (PHQ-9) shows satisfactory psychometric properties for the measurement of depressive symptoms [Stage 2] by Arish Mudra Rakshasa-Loots, Thandi Hamana, Busiswa Fanqa, Filicity Lindani, Kaylee van Wyhe, Sharon Kruger and Barbara Laughton in Brain and Neuroscience Advances

sj-csv-8-bna-10.1177_23982128231194452 – Supplemental material for isiXhosa translation of the Patient Health Questionnaire (PHQ-9) shows satisfactory psychometric properties for the measurement of depressive symptoms [Stage 2] Click here for additional data file. Supplemental material, sj-csv-8-bna-10.1177_23982128231194452 for isiXhosa translation of the Patient Health Questionnaire (PHQ-9) shows satisfactory psychometric properties for the measurement of depressive symptoms [Stage 2] by Arish Mudra Rakshasa-Loots, Thandi Hamana, Busiswa Fanqa, Filicity Lindani, Kaylee van Wyhe, Sharon Kruger and Barbara Laughton in Brain and Neuroscience Advances

sj-csv-9-bna-10.1177_23982128231194452 – Supplemental material for isiXhosa translation of the Patient Health Questionnaire (PHQ-9) shows satisfactory psychometric properties for the measurement of depressive symptoms [Stage 2] Click here for additional data file. Supplemental material, sj-csv-9-bna-10.1177_23982128231194452 for isiXhosa translation of the Patient Health Questionnaire (PHQ-9) shows satisfactory psychometric properties for the measurement of depressive symptoms [Stage 2] by Arish Mudra Rakshasa-Loots, Thandi Hamana, Busiswa Fanqa, Filicity Lindani, Kaylee van Wyhe, Sharon Kruger and Barbara Laughton in Brain and Neuroscience Advances

sj-docx-1-bna-10.1177_23982128231194452 – Supplemental material for isiXhosa translation of the Patient Health Questionnaire (PHQ-9) shows satisfactory psychometric properties for the measurement of depressive symptoms [Stage 2] Click here for additional data file. Supplemental material, sj-docx-1-bna-10.1177_23982128231194452 for isiXhosa translation of the Patient Health Questionnaire (PHQ-9) shows satisfactory psychometric properties for the measurement of depressive symptoms [Stage 2] by Arish Mudra Rakshasa-Loots, Thandi Hamana, Busiswa Fanqa, Filicity Lindani, Kaylee van Wyhe, Sharon Kruger and Barbara Laughton in Brain and Neuroscience Advances

sj-docx-2-bna-10.1177_23982128231194452 – Supplemental material for isiXhosa translation of the Patient Health Questionnaire (PHQ-9) shows satisfactory psychometric properties for the measurement of depressive symptoms [Stage 2] Click here for additional data file. Supplemental material, sj-docx-2-bna-10.1177_23982128231194452 for isiXhosa translation of the Patient Health Questionnaire (PHQ-9) shows satisfactory psychometric properties for the measurement of depressive symptoms [Stage 2] by Arish Mudra Rakshasa-Loots, Thandi Hamana, Busiswa Fanqa, Filicity Lindani, Kaylee van Wyhe, Sharon Kruger and Barbara Laughton in Brain and Neuroscience Advances

sj-docx-3-bna-10.1177_23982128231194452 – Supplemental material for isiXhosa translation of the Patient Health Questionnaire (PHQ-9) shows satisfactory psychometric properties for the measurement of depressive symptoms [Stage 2] Click here for additional data file. Supplemental material, sj-docx-3-bna-10.1177_23982128231194452 for isiXhosa translation of the Patient Health Questionnaire (PHQ-9) shows satisfactory psychometric properties for the measurement of depressive symptoms [Stage 2] by Arish Mudra Rakshasa-Loots, Thandi Hamana, Busiswa Fanqa, Filicity Lindani, Kaylee van Wyhe, Sharon Kruger and Barbara Laughton in Brain and Neuroscience Advances

sj-docx-4-bna-10.1177_23982128231194452 – Supplemental material for isiXhosa translation of the Patient Health Questionnaire (PHQ-9) shows satisfactory psychometric properties for the measurement of depressive symptoms [Stage 2] Click here for additional data file. Supplemental material, sj-docx-4-bna-10.1177_23982128231194452 for isiXhosa translation of the Patient Health Questionnaire (PHQ-9) shows satisfactory psychometric properties for the measurement of depressive symptoms [Stage 2] by Arish Mudra Rakshasa-Loots, Thandi Hamana, Busiswa Fanqa, Filicity Lindani, Kaylee van Wyhe, Sharon Kruger and Barbara Laughton in Brain and Neuroscience Advances

sj-tiff-10-bna-10.1177_23982128231194452 – Supplemental material for isiXhosa translation of the Patient Health Questionnaire (PHQ-9) shows satisfactory psychometric properties for the measurement of depressive symptoms [Stage 2] Click here for additional data file. Supplemental material, sj-tiff-10-bna-10.1177_23982128231194452 for isiXhosa translation of the Patient Health Questionnaire (PHQ-9) shows satisfactory psychometric properties for the measurement of depressive symptoms [Stage 2] by Arish Mudra Rakshasa-Loots, Thandi Hamana, Busiswa Fanqa, Filicity Lindani, Kaylee van Wyhe, Sharon Kruger and Barbara Laughton in Brain and Neuroscience Advances

sj-tiff-11-bna-10.1177_23982128231194452 – Supplemental material for isiXhosa translation of the Patient Health Questionnaire (PHQ-9) shows satisfactory psychometric properties for the measurement of depressive symptoms [Stage 2] Click here for additional data file. Supplemental material, sj-tiff-11-bna-10.1177_23982128231194452 for isiXhosa translation of the Patient Health Questionnaire (PHQ-9) shows satisfactory psychometric properties for the measurement of depressive symptoms [Stage 2] by Arish Mudra Rakshasa-Loots, Thandi Hamana, Busiswa Fanqa, Filicity Lindani, Kaylee van Wyhe, Sharon Kruger and Barbara Laughton in Brain and Neuroscience Advances

sj-tiff-12-bna-10.1177_23982128231194452 – Supplemental material for isiXhosa translation of the Patient Health Questionnaire (PHQ-9) shows satisfactory psychometric properties for the measurement of depressive symptoms [Stage 2] Click here for additional data file. Supplemental material, sj-tiff-12-bna-10.1177_23982128231194452 for isiXhosa translation of the Patient Health Questionnaire (PHQ-9) shows satisfactory psychometric properties for the measurement of depressive symptoms [Stage 2] by Arish Mudra Rakshasa-Loots, Thandi Hamana, Busiswa Fanqa, Filicity Lindani, Kaylee van Wyhe, Sharon Kruger and Barbara Laughton in Brain and Neuroscience Advances
