# Supplementary material for: isiXhosa translation of the Patient Health Questionnaire (PHQ-9) shows satisfactory psychometric properties for the measurement of depressive symptoms [Stage 2]
Source: Brain Neurosci Adv. 2023 Aug 31;7:23982128231194452. doi: 10.1177/23982128231194452 (PMC10475240; doi:10.1177/23982128231194452)
Supplement: sj-docx-1-bna-10.1177_23982128231194452 – Supplemental material for isiXhosa translation of the Patient Health Questionnaire (PHQ-9) shows satisfactory psychometric properties for the measurement of depressive symptoms [Stage 2] [file sj-docx-1-bna-10.1177_23982128231194452.docx]

**GOLD DEPRESSION STUDY**

Please answer these questions as honestly as possible. Your answers are confidential, and **we will never share your answers with anyone without your permission.**

**Participant # _____________________________ Date ______________**

1. What is your age?

|  |
| --- |

2. What is your grade at school?

|  |
| --- |

3. What is your gender?

| Boy | Girl | Trans/Genderqueer |
| --- | --- | --- |
| Other (please describe): | | |

4. What is your race or ethnicity?

| Black/African | White/Caucasian | Coloured |
| --- | --- | --- |
| Other (please describe): | | |

5. What is your sexual orientation?

| Straight/Heterosexual | Gay/Lesbian/Homosexual | Bisexual/Pansexual |
| --- | --- | --- |
| Other (please describe): | | |

6. In the last six months, did you drink any alcohol?

| Yes | No |
| --- | --- |
| If yes, how much alcohol do you usually drink in a week: | |

7. In the last six months, did you smoke any cigarettes?

| Yes | No |
| --- | --- |
| If yes, how many cigarettes do you usually smoke in a day: | |

8. In the last six months, did you take any recreational drugs (dagga, tik, mandrax, marijuana, ecstasy, cocaine, etc.)?

| Yes | No |
| --- | --- |
