# Supplementary material for: isiXhosa translation of the Patient Health Questionnaire (PHQ-9) shows satisfactory psychometric properties for the measurement of depressive symptoms [Stage 2]
Source: Brain Neurosci Adv. 2023 Aug 31;7:23982128231194452. doi: 10.1177/23982128231194452 (PMC10475240; doi:10.1177/23982128231194452)
Supplement: sj-docx-2-bna-10.1177_23982128231194452 – Supplemental material for isiXhosa translation of the Patient Health Questionnaire (PHQ-9) shows satisfactory psychometric properties for the measurement of depressive symptoms [Stage 2] [file sj-docx-2-bna-10.1177_23982128231194452.docx]

**UPHONONONGO LOKUDAKUMBA LWE-GOLD**

Nceda phendula le mibuzo ngokunyanisekileyo kangangoko. Iimpendulo zakho ziyimfihlo, **kwaye asoze sabelane ngeempendulo zakho naye nabani na ngaphandle kwemvume yakho.**

**Umthabathinxaxheba # ______________________ Umhla _____________**

1. Mingaphi iminyaka yakho?

|  |
| --- |

2. Ufunda kweliphi ibanga esikolweni?

|  |
| --- |

3. Sesiphi isini sakho?

| Inkwenkwe | Intombazana | Umntu otshintshe isini |
| --- | --- | --- |
| Okunye (nceda chaza): | | |

4. Uloluphi uhlanga?

| Umntu oNtsundu / umAfrika | Umntu / oMhlophe | iKhaladi |
| --- | --- | --- |
| Okunye (nceda chaza): | | |

5. Uzinxulumanisa nesiphi isini?

| Ndingothandana nabasini sahlukileyo kwesam | Ndiyigeyi / Ndiyilesbhiyeni / Ndingothandana nabasini sinye nesam | Ndingothandana nabazini ezibini ezahlukileyo |
| --- | --- | --- |
|  | | |

6. Kwiinyanga ezintandathu (6) ezidlulileyo, uke wasela nabuphi na utywala?

| Ewe | Hayi |
| --- | --- |
| Ukuba ngu-ewe: butywala obungakanani oqhele ubusela ngeveki: | |

7. Kwiinyanga ezintandathu (6) ezidlulileyo, ingaba ukhe walitshaya na icuba?

| Ewe | Hayi |
| --- | --- |
| Ukuba uyavuma, uqhele ukutshaya icuba elingakane ngosuku: | |

8. Kwiinyanga ezintandathu ezidlulileyo, ingaba ukhe wasebenzisa naziphi na iziyobisi zokuzonwabisa (intsangu, ithikhi, imandraksi, iekhstesi, ikhokheyini, njl.njl.)?

| Ewe | Hayi |
| --- | --- |
