# Supplementary material for: isiXhosa translation of the Patient Health Questionnaire (PHQ-9) shows satisfactory psychometric properties for the measurement of depressive symptoms [Stage 2]
Source: Brain Neurosci Adv. 2023 Aug 31;7:23982128231194452. doi: 10.1177/23982128231194452 (PMC10475240; doi:10.1177/23982128231194452)
Supplement: sj-docx-3-bna-10.1177_23982128231194452 – Supplemental material for isiXhosa translation of the Patient Health Questionnaire (PHQ-9) shows satisfactory psychometric properties for the measurement of depressive symptoms [Stage 2] [file sj-docx-3-bna-10.1177_23982128231194452.docx]

**PATIENT HEALTH QUESTIONNAIRE (PHQ-9)**

**Participant # _____________________________ Date ______________**

| **Over the *last 2 weeks*, how often have you been bothered by any of the following problems?** *(use “✓” to indicate your answer)* | **Not at all** | **Several days** | **More than half the days** | **Nearly every day** |
| --- | --- | --- | --- | --- |
| **1.** Little interest or pleasure in doing things | 0 | 1 | 2 | 3 |
| **2.** Feeling down, depressed, or hopeless | 0 | 1 | 2 | 3 |
| **3.** Trouble falling or staying asleep, or sleeping too much | 0 | 1 | 2 | 3 |
| **4.** Feeling tired or having little energy | 0 | 1 | 2 | 3 |
| **5.** Poor appetite or overeating | 0 | 1 | 2 | 3 |
| **6.** Feeling bad about yourself – or that you are a failure or have let yourself or your family down | 0 | 1 | 2 | 3 |
| **7.** Trouble concentrating on things, such as reading or watching television | 0 | 1 | 2 | 3 |
| **8.** Moving or speaking so slowly that other people could have noticed? Or the opposite – being so fidgety or restless that you have been moving around a lot more than usual | 0 | 1 | 2 | 3 |
| **9.** Thoughts that you would be better off dead or of hurting yourself in some way | 0 | 1 | 2 | 3 |

**All Nine Items Completed? Y / N For office coding** **_____ + _____ + _____ + _____**

**= Total Score: _____**

| **If you have chosen a number higher than 0 for any problems, how difficult have these problems made it for you to do your work, take care of things at home, or get along with other people?** | | | |
| --- | --- | --- | --- |
| **Not difficult at all** | **Somewhat difficult** | **Very difficult** | **Extremely difficult** |
|  |  |  |  |
