# Supplementary material for: isiXhosa translation of the Patient Health Questionnaire (PHQ-9) shows satisfactory psychometric properties for the measurement of depressive symptoms [Stage 2]
Source: Brain Neurosci Adv. 2023 Aug 31;7:23982128231194452. doi: 10.1177/23982128231194452 (PMC10475240; doi:10.1177/23982128231194452)
Supplement: sj-docx-4-bna-10.1177_23982128231194452 – Supplemental material for isiXhosa translation of the Patient Health Questionnaire (PHQ-9) shows satisfactory psychometric properties for the measurement of depressive symptoms [Stage 2] [file sj-docx-4-bna-10.1177_23982128231194452.docx]

**IPHEPHA LEMIBUZO LEMPILO YESIGULANE
(PATIENT HEALTH QUESTIONNAIRE) (PHQ-9)**

**Umthabathinxaxheba # ______________________ Umhla _____________**

| **Kwiiveki ezi-2 ezidlulileyo, ingaba uhlutshwe rhoqo kangakanani yiyo nayiphi na kwezi ngxaki zilandelayo?** *(sebenzisa “✓” ukubonisa impendulo yakho)* | **Andikhange konke konke** | **Iintsuku eziliqela** | **Ngaphezulu kwesiqingatha seentsuk** | **Phantse yonke imihl** |
| --- | --- | --- | --- | --- |
| **1.** Umdla okanye ubumnandi obuncinci ekwenzeni izinto | 0 | 1 | 2 | 3 |
| **2.** Ukuziva unomoya ophantsi, udakumbile, okanye ungenathemba | 0 | 1 | 2 | 3 |
| **3.** Ukusokola ukulala, okanye ukulala ngokugqithisileyo | 0 | 1 | 2 | 3 |
| **4.** Ukuziva udiniwe okanye ungenamandla kangako | 0 | 1 | 2 | 3 |
| **5.** Ukungacaceli ukutya okanye ukutya ngokugqithisileyo | 0 | 1 | 2 | 3 |
| **6.** Ukuziva kakubi ngawe – okanye ukuba awuphumeleli okanye udanise wena okanye udanise usapho lwakho | 0 | 1 | 2 | 3 |
| **7.** Ingxaki yokuzikisa ingqondo ezintweni, ezifana nokufunda okanye ukubukela umabonakude | 0 | 1 | 2 | 3 |
| **8.** Ukuhamba okanye ukuthetha ngokucothayo kangangokuba abanye abantu babe bakuqaphele oko? Okanye okuchaseneyo noko – ukungqunga okanye ukungazinzi kangangokuba uzulazule kakhulu kunokuqhelekileyo | 0 | 1 | 2 | 3 |
| **9.** Iingcinga zokuba kungcono ubhubhe okanye ukuzenzakalisa ngandlela ithile | 0 | 1 | 2 | 3 |

**ZONKE IZINTO EZILITHOBA ZIGQIBEKILE? UKUFAKWA IKHOWUDI YIOFISI:** **_____ + _____ + _____ + _____**

**EWE / HAYI = Inqaku Lilonke: _________**

| **Ukuba ngaba ukhethe inani elingaphezulu kwe-0 ngazo naziphi iingxaki, ezi ngxaki zenze kwanzima kangakanani kuwe ukuba wenze umsebenzi wakho, ukulungisa izinto ekhaya, okanye ukuvana nabanye abantu** | | | |
| --- | --- | --- | --- |
| **Akunzimanga konke konke** | **Kunzinyana noko** | **Kunzima kakhulu** | **Kube nzima ngokugqithisileyo** |
|  |  |  |  |
