# Supplementary figures and images for: isiXhosa translation of the Patient Health Questionnaire (PHQ-9) shows satisfactory psychometric properties for the measurement of depressive symptoms [Stage 2]
Source: Brain Neurosci Adv. 2023 Aug 31;7:23982128231194452. doi: 10.1177/23982128231194452 (PMC10475240; doi:10.1177/23982128231194452)

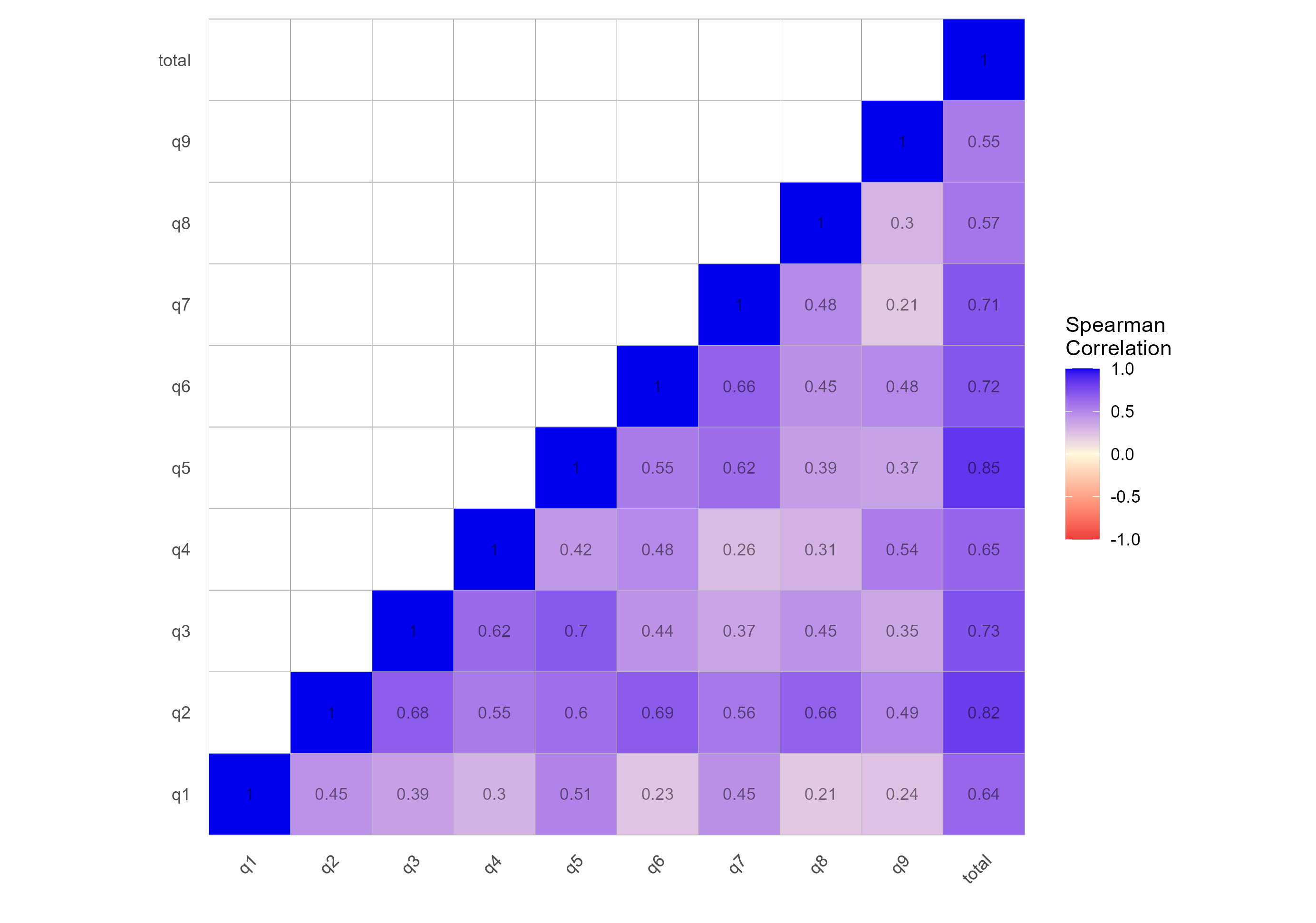

Supplement: sj-tiff-10-bna-10.1177_23982128231194452 – Supplemental material for isiXhosa translation of the Patient Health Questionnaire (PHQ-9) shows satisfactory psychometric properties for the measurement of depressive symptoms [Stage 2] [file sj-tiff-10-bna-10.1177_23982128231194452.tiff]

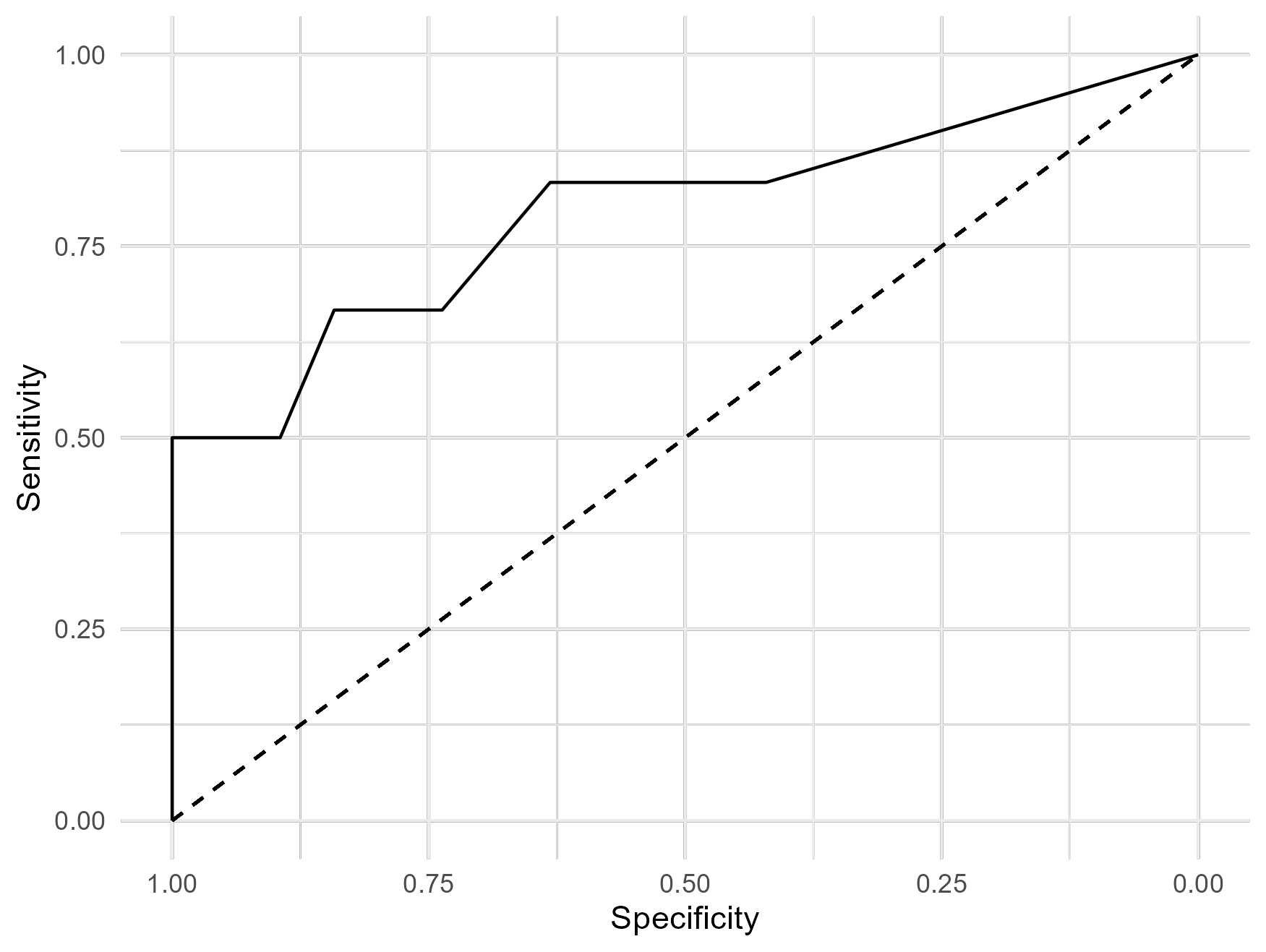

Supplement: sj-tiff-11-bna-10.1177_23982128231194452 – Supplemental material for isiXhosa translation of the Patient Health Questionnaire (PHQ-9) shows satisfactory psychometric properties for the measurement of depressive symptoms [Stage 2] [file sj-tiff-11-bna-10.1177_23982128231194452.tiff]

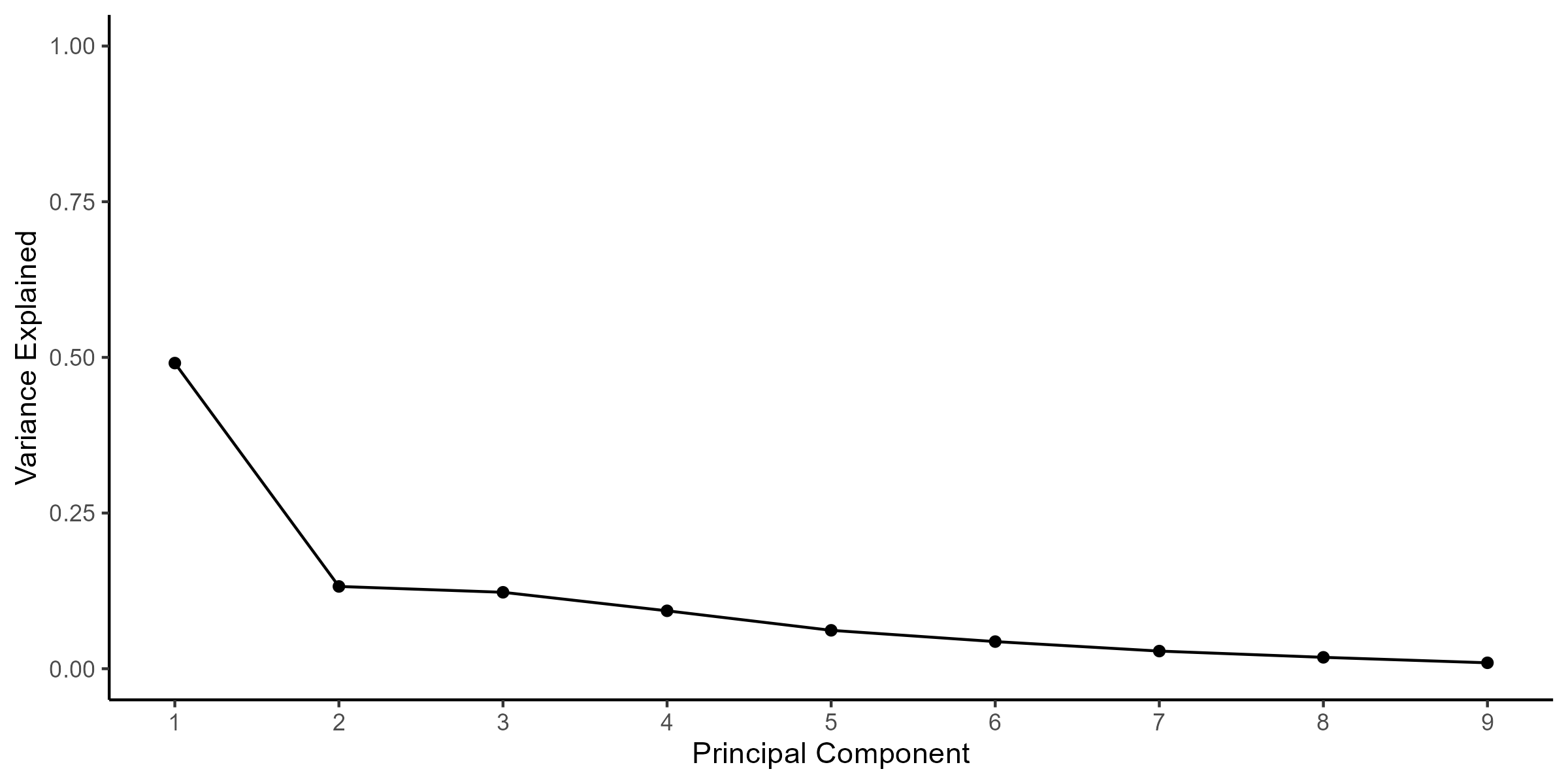

Supplement: sj-tiff-12-bna-10.1177_23982128231194452 – Supplemental material for isiXhosa translation of the Patient Health Questionnaire (PHQ-9) shows satisfactory psychometric properties for the measurement of depressive symptoms [Stage 2] [file sj-tiff-12-bna-10.1177_23982128231194452.tiff]
